# Supplementary material for: Acetylcholine receptor based chemogenetics engineered for neuronal inhibition and seizure control assessed in mice
Source: Nat Commun. 2024 Jan 18;15:601. doi: 10.1038/s41467-024-44853-8 (PMC10796428; doi:10.1038/s41467-024-44853-8)
Supplement: Supplementary file 1 — Supplementary Information [file 41467_2024_44853_MOESM1_ESM.pdf]

Supplementary Information for

Acetylcholine receptor based chemogenetics engineered for neuronal inhibition and seizure control assessed in mice

Quynh-Anh Nguyen*[a,1], Peter M. Klein*[a,1], Cheng Xie[b], Katelyn N. Benthall[b], Jillian Iafrati[b], Jesslyn Homidan[a], Jacob T. Bendor[b], Barna Dudok[a,c], Jordan S. Farrell[a], Tilo Gschwind[a], Charlotte L. Porter [a], Annahita Keravala[b], G. Steven Dodson[b] and Ivan Soltesz[a]

[a] Department of Neurosurgery, Stanford University, Stanford, CA 94305

[b] CODA Biotherapeutics, 240 East Grand Ave. South San Francisco, CA 94080

[c] Department of Neurology, Baylor College of Medicine, Houston, TX 77030

[1] These authors contributed equally

*Corresponding authors: Quynh-Anh Nguyen (qanguyen@stanford.edu, 650-723-1168) and Peter M. Klein (kleinp@stanford.edu, 650-725-9055); 1201 Welch Rd, Palo Alto, CA 94305

```
Signal peptide 1-22
MRCSPGGVWL ALAASLLHVS LQGEFQRKLY KELVKNYNPL ERPVANDSQP   50
α7nAChR LBD

LTVYFSLSLL QIMDVDEKNQ VLTTNIWLQM SWTDHYLQWN VSEYPGVKTV  100
α7nAChR LBD

RFPDGQIWKP DILLYNSADE RFDATFHTNV LVNSSGHCQY LPPGIFKSSC  150
α7nAChR LBD

α1GlyR Cys loop
PMDLKNFPMD VQTCKLKFGS WSYGGWSLDL QMQEADISGY IPNGEWDLVG  200
α7nAChR LBD

Pre-M1 linker
IPGKRSERFY ECCKEPYPDV TFTVTMRRRM GYYLIQMYIP SLLIVILSWI  250
α7nAChR LBD                      α1GlyR IPD

SFWINMDAAP ARVGLGITTV LTMTTQSSGS RASLPKVSYV KAIDIWMAVC  300
α1GlyR IPD

α1Ins sequence
LLFVFSALLE YAAVNFVSRQ HKELLRFRRK RRHHKSPMLN LFQEDEAGEG  350
α1GlyR IPD

RFNFSAYGMG PACLQAKDGI SVKGANNSNT TNPPPAPSKS PEEMRKLFIQ  400
α1GlyR IPD

RAKKIDKISR IGFPMAFLIF NMFYWIIYKI VRREDVHNQ              439
α1GlyR IPD
```

Supplementary Fig. 1: Amino acid sequence of the BARNI channel.

Sequences for α7nAChR ligand binding domain (LBD) and α1GlyR ion pore domain (IPD) elements are labeled, while additional amino acid sequences of interest are highlighted in blue. All differences from the parent α7/Gly chimeric receptor (Grutter et al., 2005), which was also used as the shared base for the PSAM eLGICs (Magnus et al., 2011 and Magnus et al., 2019), are labeled in red.

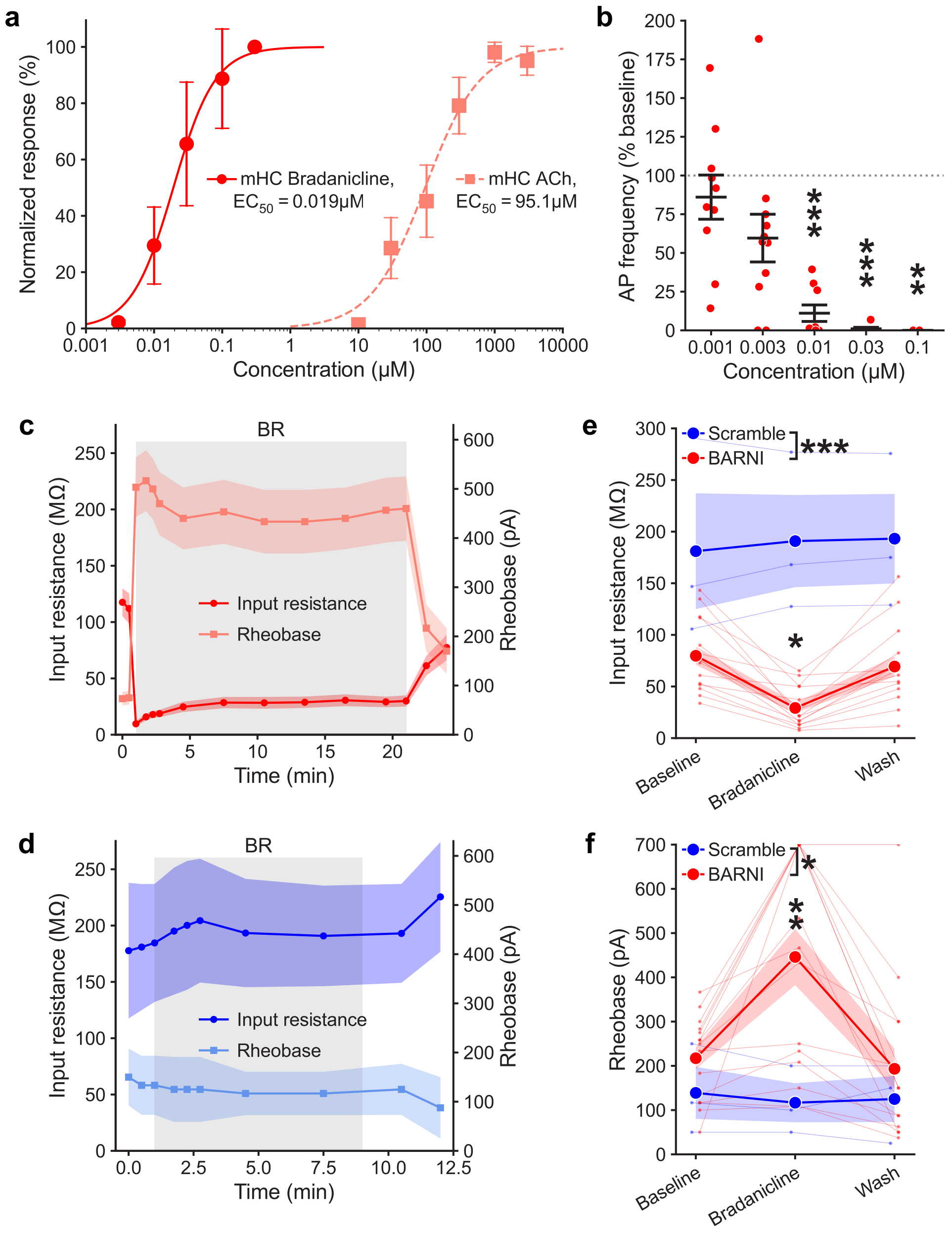

Supplementary Fig. 2: Validation of BARNI channel efficacy in dissociated hippocampal neurons.

All data is from current clamp recordings of dissociated and cultured P0 mouse hippocampal (mHC) neurons at DIV13-16. **a)** Dose-response relationship during application of bradanicline (circles) or ACh (squares), including non-linear regression fits used to calculate EC50 responses for each drug. **b)** Spontaneous action potential (AP) firing rates, relative to normalized baseline firing activity for each cell, show dose-dependent decreases across a similar range of bradanicline concentrations as panel A ($F(5,44)=14.37$, $P<0.001$; 0.001μM: $P=0.921$; 0.003μM: $P=0.058$; 0.01μM: $P<0.001$; 0.03μM: $P<0.001$; 0.1μM: $P=0.003$). **c)** BARNI-expressing mHC neurons display durable decreases in input resistance and increases in rheobase currents during a 20 min application of 0.3 μM bradanicline (BR). **d)** Scramble-expressing neurons showed no changes in measured intrinsic properties during an 8 min drug application. **e)** Time-binned input resistance values for individual animals (small circles) and within groups (large circles) show lower values in BARNI-expressing vs Scramble-expressing neurons ($F(1,48)=71.70$, $P<0.001$), with decreases relative to baseline specifically after bradanicline application ($F(2,48)=4.88$, Bradanicline: $P=0.015$, Wash: $P=0.872$). **f)** Time-binned rheobase values were higher in BARNI vs Scramble-expressing neurons ($F(1,48)=6.21$, $P=0.016$), with elevated values relative to baseline during bradanicline application ($F(2,48)=7.82$, Bradanicline: $P=0.006$, Wash: $P=0.900$). Baseline values are averaged across 1 min prior to drug application, bradanicline values are from the final 5 min of application and Wash values are from <3 min after drug. Mean ± SEM, A: 2-8 replicates per condition; B: n = 11 neurons; C-F: n = 15 BARNI, 3 Scramble neurons. *$P<0.05$, **$P<0.01$, ***$P<0.001$ (one- or two-way ANOVA with two-sided Tukey's HSD). Source data are provided as a Source Data file.

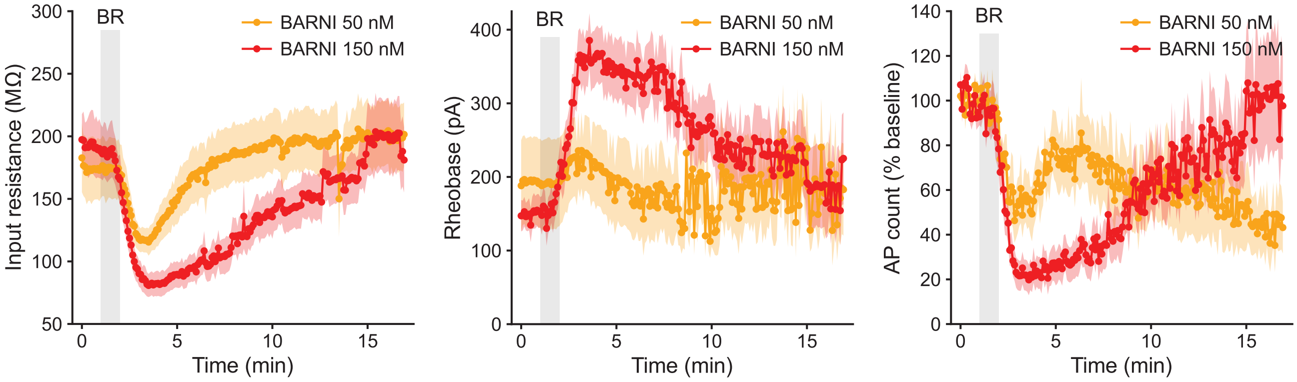

Supplementary Fig. 3: BARNI channel activation dose response in acute hippocampal slices.

Time course of BARNI-expressing CA1 neuron responses to a 1 min bath application of varied bradanicline (BR) doses in acute hippocampal slices. Compared to 150 nM bradanicline responses (also shown in Fig. 1), a lower 50 nM bradanicline dose produced smaller amplitude shifts in neuronal input resistance, rheobase currents and action potential (AP) counts. Mean ± SEM, n = 19 cells/8 animals 150 nM, n = 7 cell/4 animals 50 nM. Source data are provided as a Source Data file.

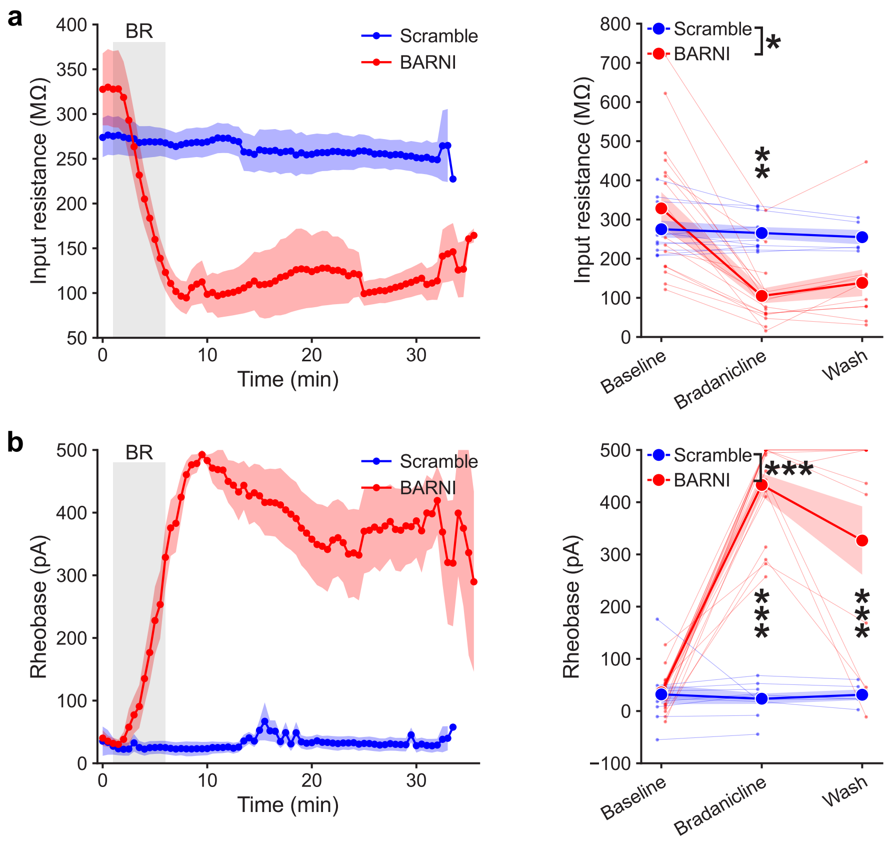

Supplementary Fig. 4: Corroboration of BARNI channel mediated suppression of neuronal excitability.

Current clamp recordings of transduced CA1 neurons in acute hippocampal slices performed in a separate cohort of animals by CODA researchers, recorded from both BARNI and Scramble-expressing cells. **a)** *Left*, time course of input resistance changes, measured with -100pA current steps, in response to 5 min bath application of bradanicline (BR; 0.15 μM), sampled every 30 sec. *Right*, Time-binned input resistance values for individual animals (small circles) and within groups (large circles) show a greater decrease in BARNI vs Scramble-expressing neurons ($F(1,64)=6.04$, $P=0.017$), occurring specifically after bradanicline application ($F(2,64)=6.46$, Baseline: $P=0.803$, Bradanicline: $P=0.006$, Wash: $P=0.368$). **b)** *Left*, time course of changing rheobase currents required to evoke action potentials in response to 5 min bath application of bradanicline. *Right*, Time-binned rheobase values show a rise in BARNI vs Scramble-expressing neurons ($F(1,64)=78.61$, $P<0.001$), that occurred after bradanicline application and persisted into the wash phase ($F(2,64)=6.05$, Baseline: $P=0.900$, Bradanicline: $P<0.001$, Wash: $P<0.001$). Baseline values are averaged across 1 minute prior to drug application, bradanicline values are 1-6 minutes after application and Wash values are > 15 minutes after drug. Mean ± SEM, n = 17 BARNI, 10 Scramble neurons. *P<0.05, **P<0.01 and ***P<0.001 (two-way ANOVA with two-sided Tukey's HSD). Source data are provided as a Source Data file.

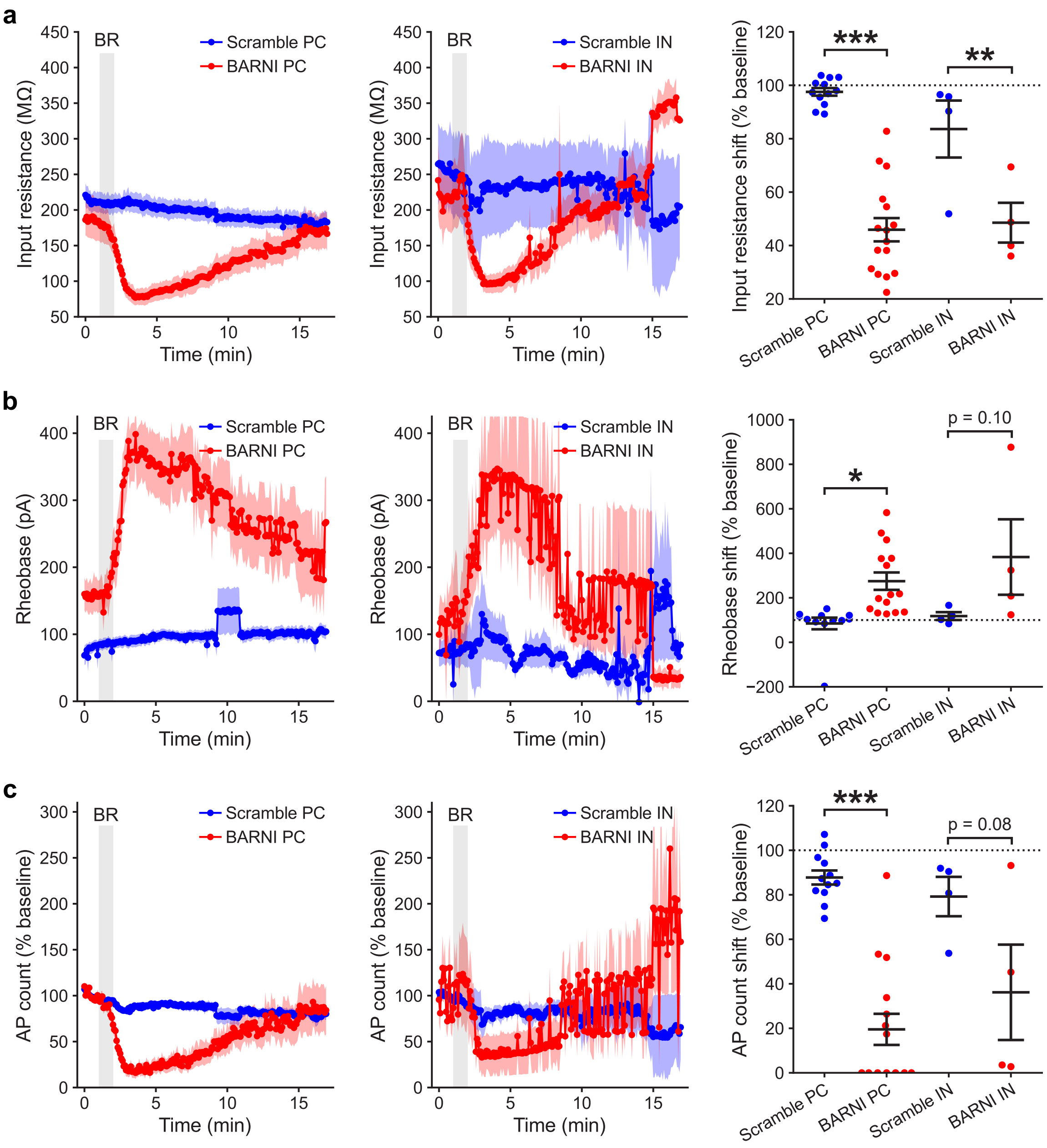

Supplementary Fig. 5: BARNI channel responses among putative CA1 neuron populations.

The overall population of CA1 neurons in acute hippocampal slices presented in Fig. 1 was divided into groups of putative pyramidal cells (PC) or interneurons (IN) that had action potential widths of 1 ms or less. **a)** Time course of input resistance changes in BARNI- and Scramble-expressing cells in response to 1 min bath application of bradanicline (BR; 0.15 µM), sampled every 5 sec. Right, input resistance values were altered after bradanicline application ($F(3,32)=32.28$, $P<0.001$), decreasing in both the BARNI PC ($P<0.001$) and BARNI IN ($P=0.010$) groups relative to their respective population of Scramble neurons. **b)** Time course of changing rheobase currents required to evoke action potentials in response to bradanicline. Right, rheobase currents shifted after bradanicline application ($F(3,31)=5.50$. $P=0.004$), increasing between BARNI relative to Scramble PCs ($P=0.019$), but not between IN populations ($P=0.099$). **c)** Time course of changes in action potential (AP) counts, normalized to the pre-drug average for each neuron, in response to bradanicline. Right, action potential counts were modulated by bradanicline application ($F(3,31)=20.15$, $P<0.001$), decreasing between BARNI relative to Scramble PCs ($P<0.001$), but not between IN populations ($P=0.076$). No differences were observed between BARNI PC and BARNI IN groups in a-c ($P=0.988$, $P=0.612$, $P=0.615$, respectively). Shifts in values for each neuron are calculated as the average within the 1-3 minutes after drug application relative to the 1 minute of baseline recording. No differences were observed between BARNI PC and BARNI IN responses. Data are presented as Mean ± SEM. n = 12 cell/7 animals Scramble PC, n = 16 cells/7 animals BARNI PC, n = 4 cell/3 animals Scramble IN, n = 4 cells/4 animals BARNI IN. *$P<0.05$, **$P<0.01$ and ***$P<0.001$ (one-way ANOVA with two-sided Tukey's HSD). Source data are provided as a Source Data file.

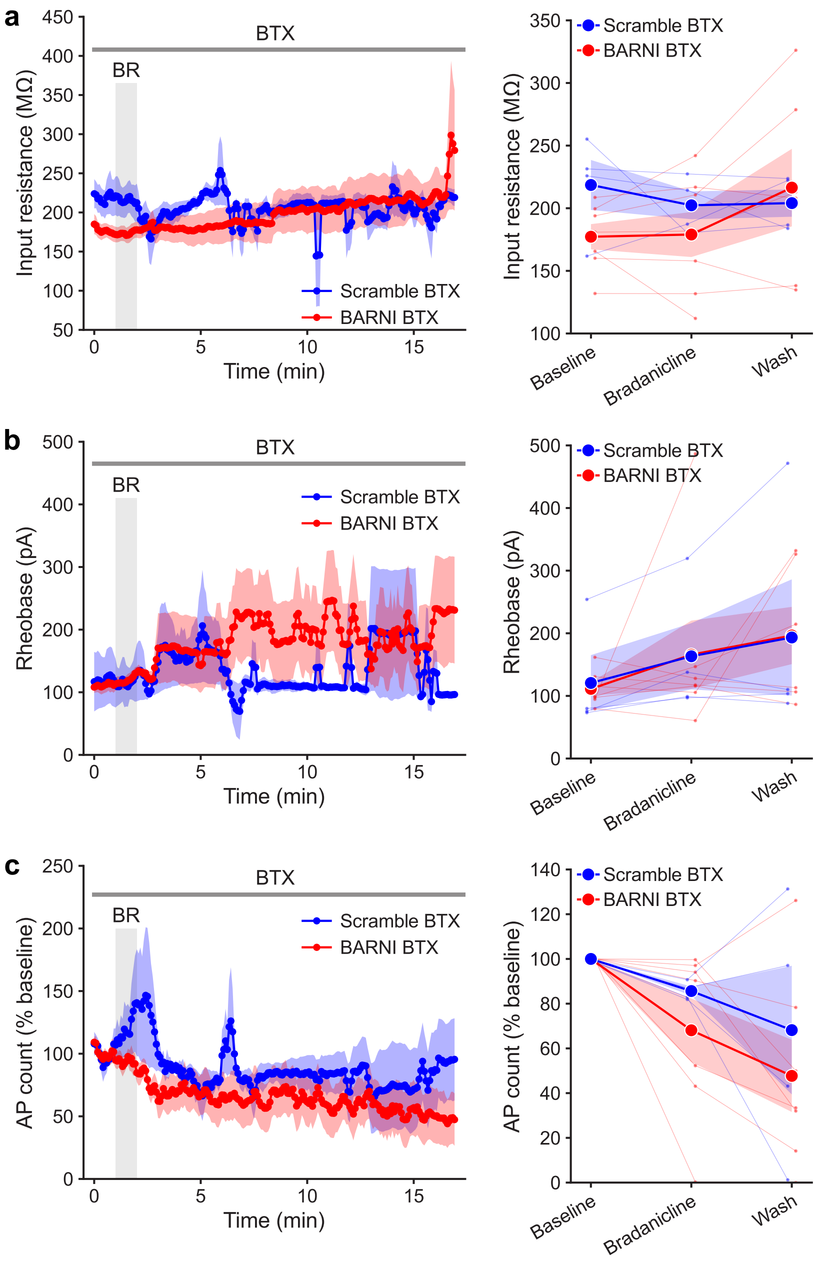

Supplementary Fig. 6: Bradanicline suppression of neuronal excitability requires receptors with α7 nACh ligand-binding domains.

Current clamp recordings of BARNI or Scramble-expressing transduced CA1 neurons in acute hippocampal slices in aCSF containing 0.25 μM α-Bungarotoxin (BTX). BTX is a selective antagonist of both endogenous α7 nAChRs and the α7 nACh ligand binding domain-containing BARNI channel. **a)** *Left*, time course of input resistance values in response to 1 min bath application of bradanicline (BR; 0.15 μM), sampled every 5 sec. *Right*, Time-binned input resistance values for individual animals (small circles) and within groups (large circles) were unchanged in BARNI or Scramble-expressing neurons by bradanicline application ($F(2,27)=0.88$, $P=0.427$). **b)** *Left*, time course of measured rheobase currents required to evoke action potentials in response to 1 min bath application of bradanicline. *Right*, Time-binned rheobase values were equivalent between BARNI vs Scramble-expressing neurons in response to bradanicline application ($F(2,26)=0.01$, $P=0.991$). **c)** *Left*, time course of action potential (AP) counts, normalized to the pre-drug average for each neuron, in response to 1 min bath application of bradanicline. *Right*, Time-binned action potential counts remained similar in BARNI vs Scramble-expressing neurons after bradanicline application ($F(2,27)=0.29$, $P=0.753$). Baseline values are averaged across 1 minute prior to drug application, bradanicline values are 1-3 minutes after application and Wash values are > 8 minutes after drug. Mean ± SEM, n = 7 cells/2 animals BARNI, n = 4 cells/2 animals Scramble. *$P<0.0\%$ (two-way ANOVA with two-sided Tukey's HSD). Source data are provided as a Source Data file.

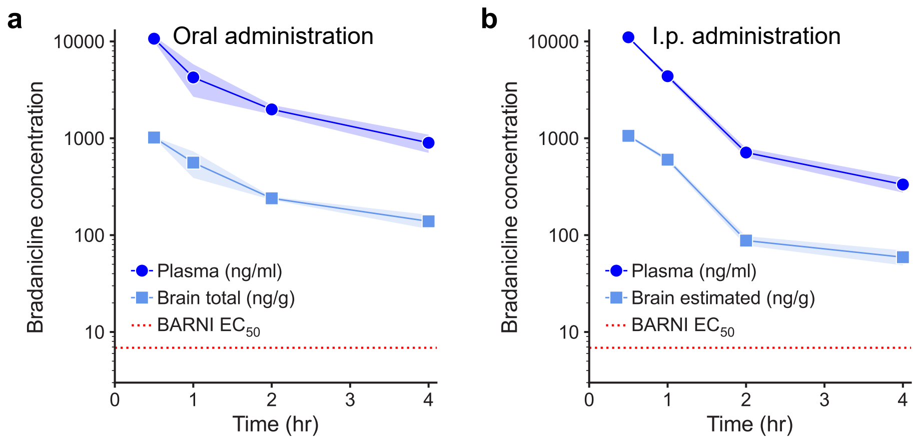

Supplementary Fig. 7: Quantifying bradanicline concentrations in plasma and brain tissue following *in vivo* dosing.

a) Measurements of bradanicline concentrations detectable in plasma (in ng/ml) and in total brain tissue (in ng/g) following an initial administration via oral gavage. **b)** Plasma concentrations of bradanicline measured after an intraperitoneal (i.p.) administration and used to calculate estimates of brain concentrations based on observed plasma-to-brain ratios following oral administration. All measurements were made using LC-MS/MS. Dotted lines in each plot indicate the EC_{50} for BARNI channel activation recorded in dissociated hippocampal neurons. Mean ± SEM, n = 3 animals per time point. Source data are provided as a Source Data file.

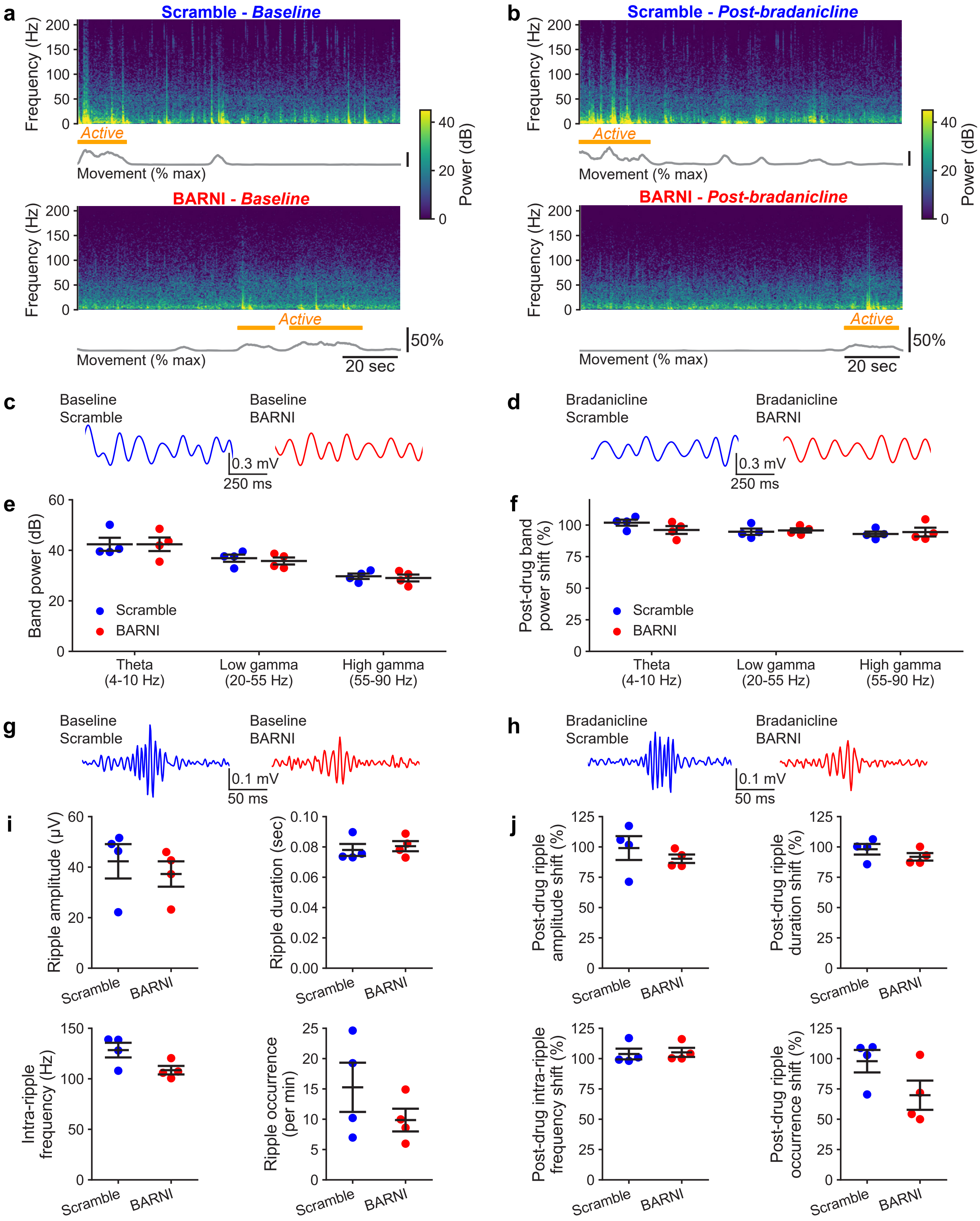

Supplementary Fig. 8: Impacts of BARNI channel on memory-associated hippocampal oscillations.

Hippocampal local field potential (LFP) was recorded with implanted 4-channel silicon probes as mice freely explored an open field. Representative LFP frequency spectra examples from Scramble- and BARNI-expressing animals during the baseline 30 min following intraperitoneal saline injection **(a)** and 30 min post-bradanicline (100 mg/kg) injection phases **(b)**. Head-mounted accelerometry tracked bouts of elevated active movement (>5 sec). Periods of theta-filtered (90-200 Hz) signal at baseline **(c)** and post-bradanicline **(d)** from active periods within the above examples. **e)** Activity-associated rhythm band power was not altered at baseline between construct groups in the theta (P=0.999), low gamma (P=0.598) or high gamma (P=0.707) frequency bands. **f)** There were also no construct-associated within animal shifts from baseline in theta (P=0.189), low gamma (P=0.744) or high gamma (P=0.718) amplitudes post-bradanicline. Representative sharp wave-ripples, filtered at 90-200 Hz, occurring during the baseline **(g)** and post-bradanicline **(h)** periods of the

above examples. **i)** At baseline, there were no differences between Scramble- and BARNI-expressing animals in the non-active LFP properties of ripple amplitude (P=0.574), duration (P=0.647), intra-ripple frequency (P=0.056) or rate of ripple occurrence (P=0.272). **j)** Post-bradanicline within animal shifts did not differ between groups in ripple amplitude (P=0.431), duration (P=0.288) or intra-ripple frequency (P=0.825), rate of ripple occurrence (P=0.115). Between group differences were assessed with two-sided t-tests. Data are presented as Mean ± SEM. n = 4 animals per group. Source data are provided as a Source Data file.

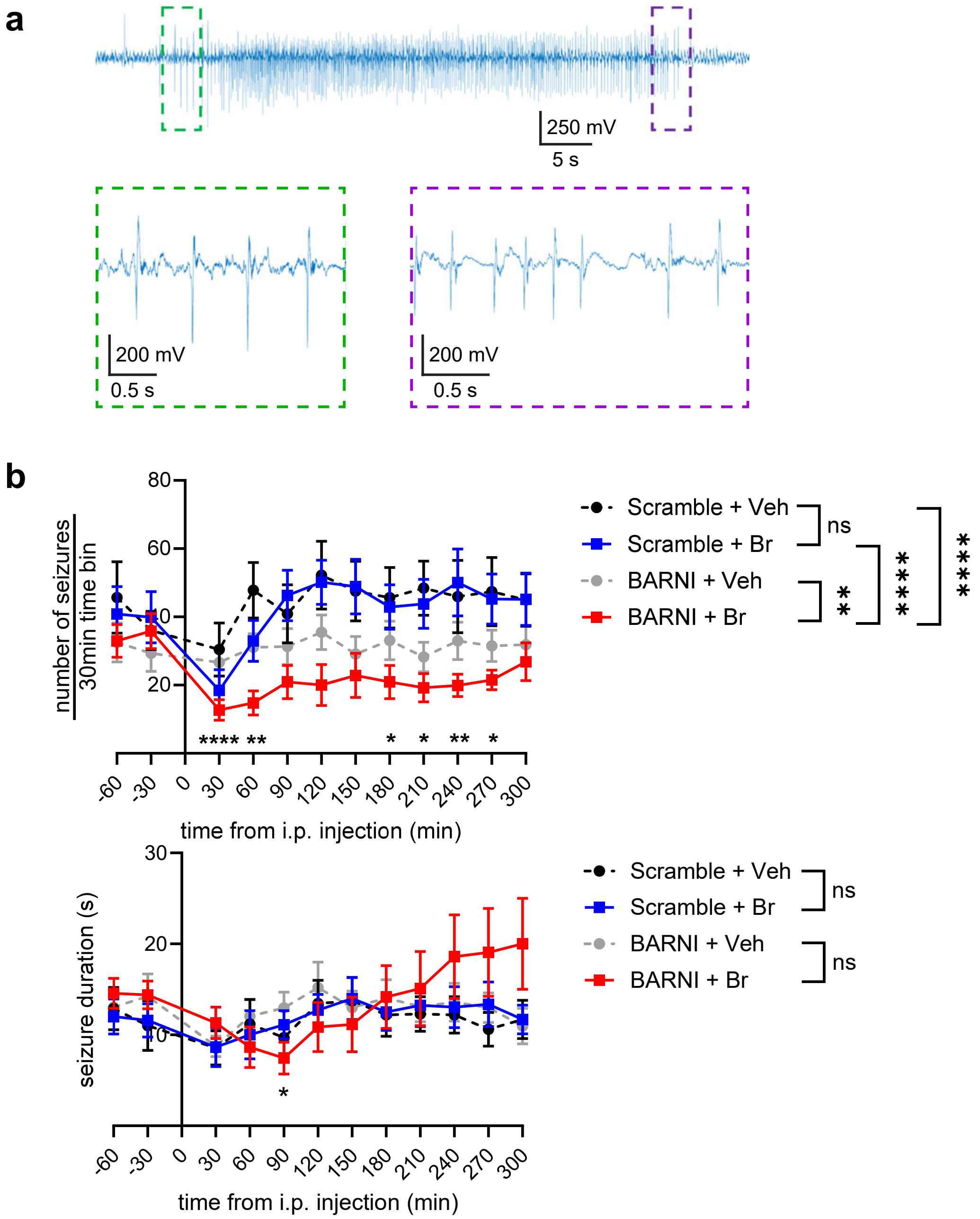

Supplementary Fig. 9: Absolute quantifications of seizure frequency and duration.

a) Representative seizure with insets showing spike frequency at the beginning (green) and towards the end (purple) of the seizure. **b)** Top, frequency of spontaneous seizures decreased after a single intraperitoneal (i.p.) injection of bradanicline (Br; 100 mg/kg). 30 minute bins. (F(33, 374)=3.322, P<0.0001, two-way RM ANOVA, Time x Vector + Drug). Bottom, brief change in the duration of spontaneous seizures observed after a single i.p. injection of bradanicline. 30 minute bins. (F(33, 374)=2.7, P<0.0001, two-way RM ANOVA, Time x Vector + Drug). Significance values on graph are shown for comparison between vehicle and bradanicline treatment in BARNI-expressing mice at each time point using paired two-sided t-test. Significance values in legend are shown for comparison between vector expression and treatment groups using a two-sided Tukey's multiple comparisons test. Mean ± SEM, n = 9 Scramble, 10 BARNI mice. *P<0.05, **P<0.01, ****P<0.0001. Source data are provided as a Source Data file.

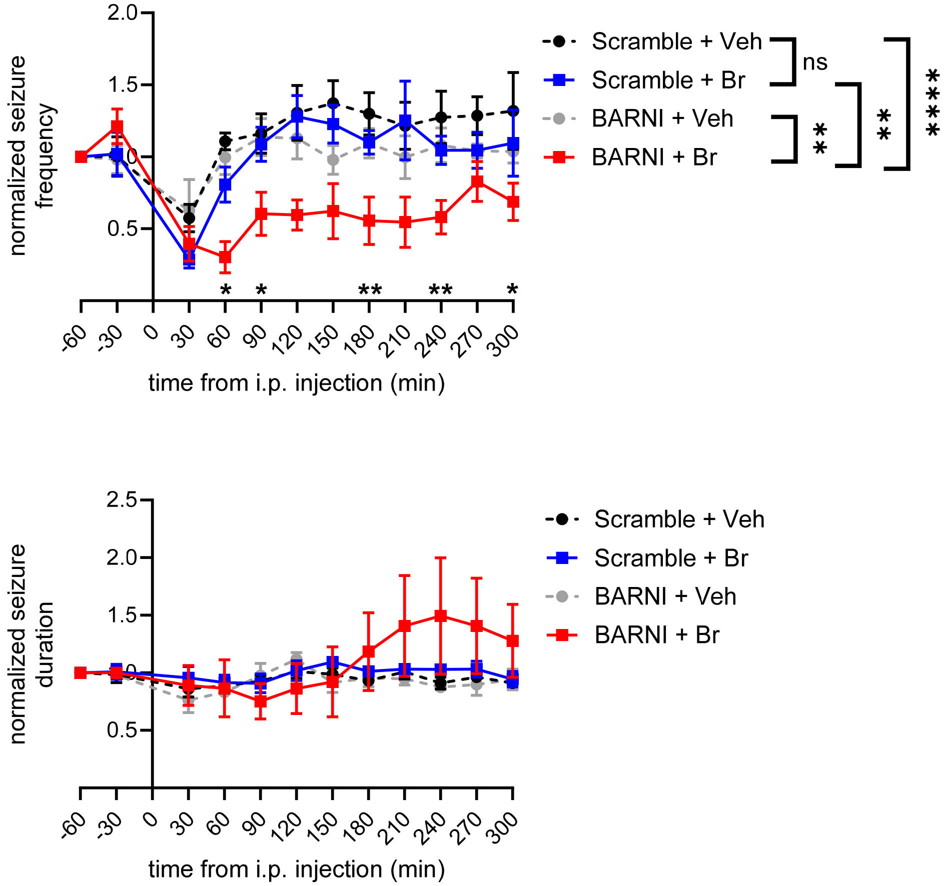

Supplementary Fig. 10: Seizure analysis using 6-second seizure duration threshold.

Top, frequency of spontaneous seizures decreased after a single intraperitoneal (i.p.) injection of bradanicline. 30 minute bins. ($F(33, 198)=1.951$, $P=0.0028$, two-way RM ANOVA, Time x Vector + Drug). *Bottom*, duration of spontaneous seizures observed after a single i.p. injection of bradanicline (Br; 100 mg/kg). 30 minute bins, dashed line indicates time of second dose. ($F(33, 198)=1.809$, $P=0.0072$, two-way RM ANOVA, Time x Vector + Drug). Significance values on graph are shown for comparison between vehicle and bradanicline treatment in BARNI-expressing mice at each time point using paired two-sided t-test. Significance values in legend are shown for comparison between vector expression and treatment groups using a two-sided Tukey's multiple comparisons test. Mean ± SEM, n = 6 Scramble, 5 BARNI mice. *$P<0.05$, **$P<0.01$, ****$P<0.0001$. Source data are provided as a Source Data file.

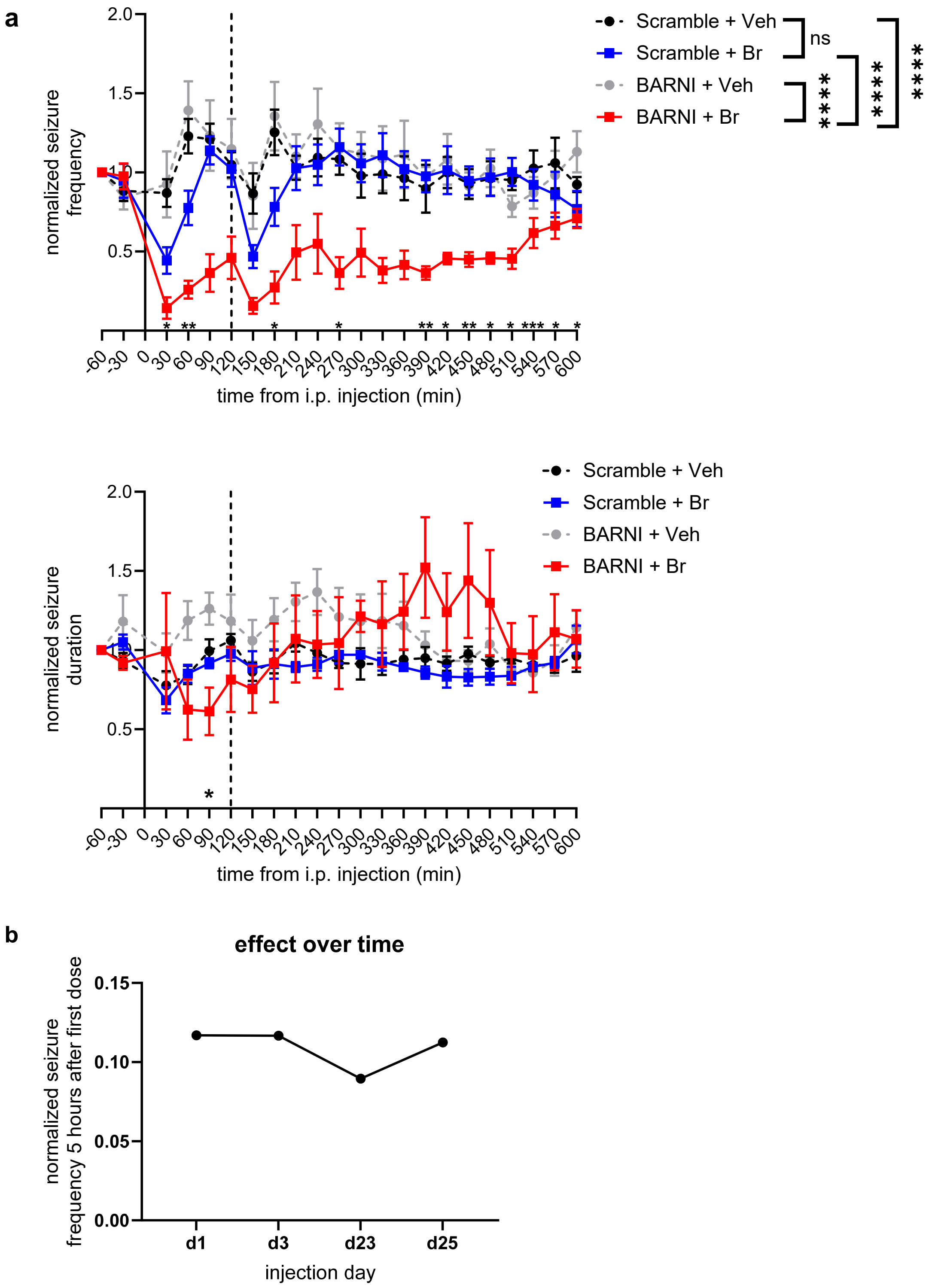

Supplementary Figure 11: Repeat dosing on seizure frequency and duration.

a) *Top*, prolonged decrease in frequency of spontaneous seizures after two intraperitoneal (i.p.) injections of the same compound of saline or bradanicline (Br; 100 mg/kg) spaced 2 hours apart. 30 minute bins, dashed line indicates time of second dose. ($F(63, 378)=4.553$, $P<0.0001$, two-way RM ANOVA, Time x Vector + Drug). *Bottom*, brief change in the duration of spontaneous seizures observed after the first of two i.p. injections of the same compound spaced 2 hours apart. 30 minute bins, dashed line indicates time of second dose. ($F(63, 378)=2.579$, $P<0.0001$, two-way RM ANOVA, Time x Vector + Drug). Significance values on graph are shown for comparison between vehicle and bradanicline treatment in BARNI-expressing mice at each time point using paired two-sided t-test. Significance values in legend are shown for comparison between vector expression and treatment groups using a two-sided Tukey's multiple comparisons test. Mean ± SEM, n = 6 Scramble, 5 BARNI mice. *$P<0.05$, **$P<0.01$, ***$P<0.001$, ****$P<0.0001$. **b)** Normalized seizure frequency 5 hours after first bradanicline dose in a 2-dose drug regimen (second dose administered 2 hours after the first) across several days in a BARNI-expressing mouse. Source data are provided as a Source Data file.

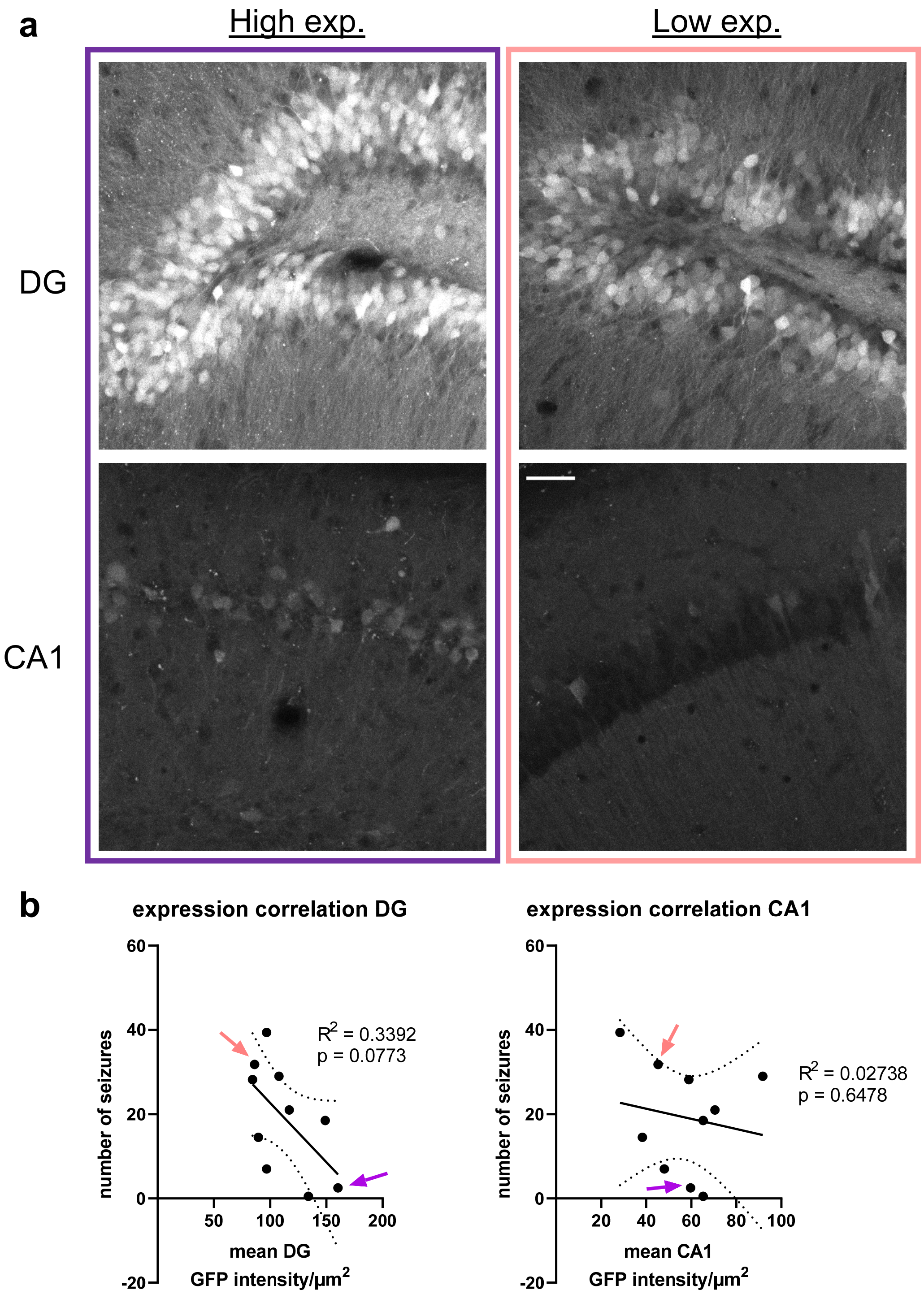

Supplementary Fig. 12: Expression of BARNI in dentate gyrus and CA1 with respect to seizure frequency.

a) Representative images of dentate gyrus (DG) and CA1 sub-regions from brain sections of high BARNI-expressing and low BARNI-expressing mice. Scale bar: 40 µm. **b)** Correlation of mean GFP intensity in either DG or CA1 with the number of seizures observed 210 minutes after injection of bradanicline in BARNI-expressing mice. Colored arrows point to respective samples in A. Dotted lines are 95% confidence bands of linear regression. n = 10 BARNI mice**.** Source data are provided as a Source Data file.

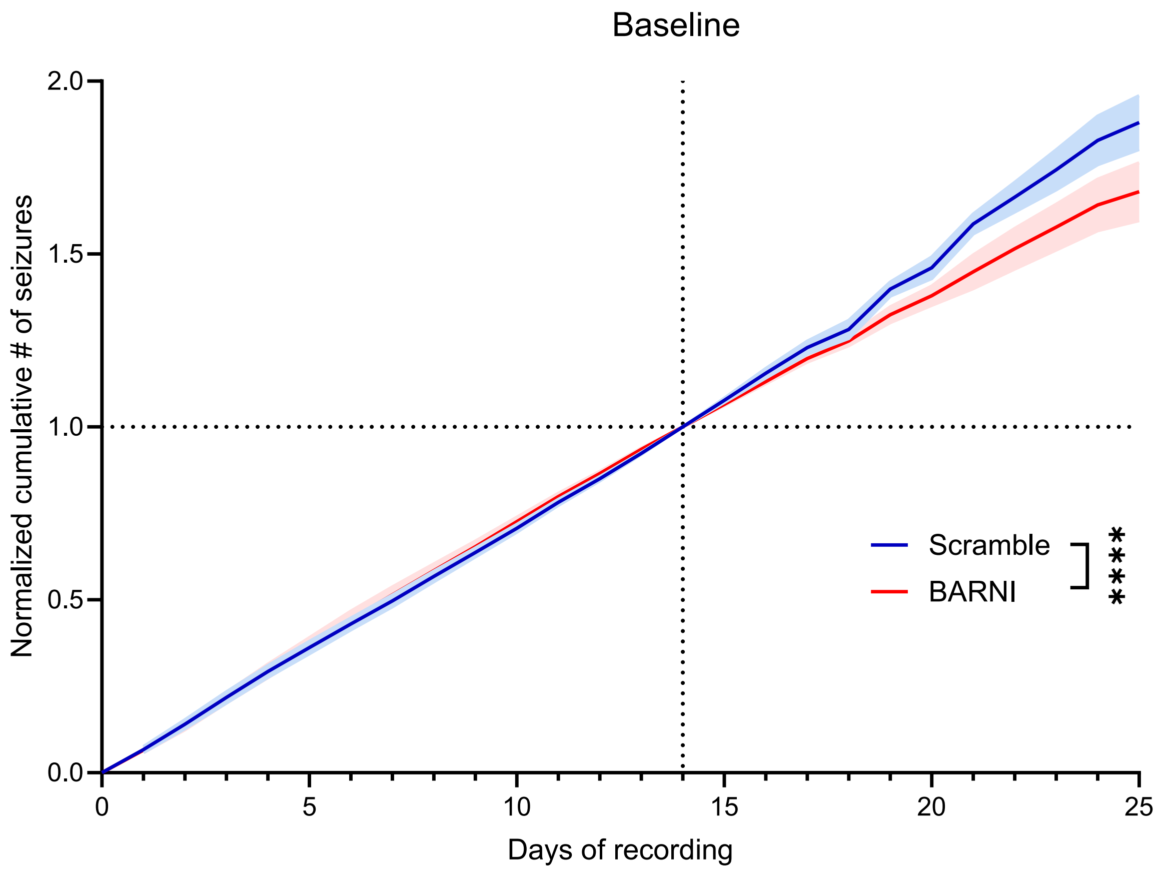

Supplementary Fig. 13: Decreasing cumulative number of seizures in BARNI-expressing mice over time.

Baseline recordings were taken starting 1-2 days after virus injection. Cumulative number of seizures over an approximately 3.5 week time period from the start of recording is shown, normalized to the cumulative number of seizures observed at 14 days of recording for each mouse. BARNI-expressing mice show a lower cumulative number of seizures at the end of the baseline recording period compared to Scramble-expressing mice (Mixed-effects model, Time x Vector: $F(24, 338) = 3.518$, $P<0.0001$). Mean ± SEM, n = 8 Scramble, 10 BARNI mice. ****$P<0.0001$. Source data are provided as a Source Data file.

Supplementary Table 1: Additional statistics related to BARNI channel activation decreasing frequency of spontaneous seizures.

Detailed statistics regarding the impact of BARNI channel activation on chronic spontaneous seizures in EEG from mice, recorded at least 3 weeks following a unilateral dorsal intrahippocampal kainic acid (IHKA) injection, as shown in Fig. 3. Changes in seizure properties were tracked in 30-minute bins, normalized to the 1 hour pre-treatment baseline for each mouse, prior to an i.p. injection of vehicle (Veh) or bradanicline (Br).

Fig. 3c Normalized seizure frequency

Tukey's multiple comparisons test	Mean Diff.	95% CI of diff.	Below threshold?	Summary	Adjusted P Value
Scramble + Vehicle vs. Scramble + Br	-0.08117	-0.3162 to 0.1539	No	ns	0.7933
Scramble + Vehicle vs. BARNI + Br	0.3643	0.1292 to 0.5993	Yes	***	0.0009
Scramble + Br vs. BARNI + Br	0.4454	0.2104 to 0.6805	Yes	****	<0.0001
BARNI + Vehicle vs. BARNI + Br	0.4857	0.2507 to 0.7208	Yes	****	<0.0001

Paired t-tests BARNI + Vehicle vs BARNI + Br

Time (min)	Below threshold?	P value	Mean of BARNI + Vehicle	Mean of BARNI + Br	Difference	SE of difference	t ratio	df
-60			1	1	0	0		
-30	No	0.152504	0.9435	1.138	-0.1949	0.1247	1.563	9
30	No	0.0536	1.262	0.3838	0.8785	0.3958	2.22	9
60	Yes	0.000556	1.129	0.4408	0.6879	0.132	5.211	9
90	No	0.126136	1.232	0.6302	0.6013	0.3567	1.686	9
120	No	0.155384	1.63	0.676	0.9537	0.615	1.551	9
150	No	0.26782	1.152	0.7296	0.4228	0.358	1.181	9
180	No	0.058892	1.251	0.6009	0.6502	0.3008	2.162	9
210	Yes	0.027401	1.063	0.5621	0.5012	0.1906	2.629	9
240	Yes	0.012084	1.169	0.6308	0.5383	0.1719	3.132	9
270	No	0.126788	1.382	0.7161	0.6657	0.3957	1.682	9
300	No	0.62223	0.9706	0.8465	0.1241	0.2433	0.5101	9

Fig. 3e Normalized seizure duration

Tukey's multiple comparisons test	Mean Diff.	95% CI of diff.	Below threshold?	Summary	Adjusted P Value
Scramble + Vehicle vs. Scramble + Br	-0.08945	-0.2791 to 0.1002	No	ns	0.5932
Scramble + Vehicle vs. BARNI + Br	-0.09487	-0.2845 to 0.09479	No	ns	0.5458
Scramble + Br vs. BARNI + Br	-0.005412	-0.1951 to 0.1842	No	ns	0.9998
BARNI + Vehicle vs. BARNI + Br	-0.06128	-0.2509 to 0.1284	No	ns	0.8239

Paired t-tests BARNI + Vehicle vs BARNI + Br

Time (min)	Below threshold?	P value	Mean of BARNI + Vehicle	Mean of BARNI + Br	Difference	SE of difference	t ratio	df
-60			1	1	0	0		
-30	No	0.649313	1.057	1.013	0.04381	0.09314	0.4703	9
30	No	0.153456	0.6751	0.8885	-0.2133	0.1369	1.559	9
60	No	0.270612	0.9165	0.7006	0.2159	0.184	1.174	9
90	Yes	0.017284	0.9887	0.5721	0.4166	0.1431	2.911	9
120	No	0.280491	1.119	0.855	0.2641	0.23	1.148	9
150	No	0.479559	0.9834	0.8243	0.1591	0.2156	0.7376	9
180	No	0.916466	1.065	1.089	-0.02408	0.2233	0.1079	9
210	No	0.667173	1.008	1.148	-0.1396	0.314	0.4445	9
240	No	0.271302	1.016	1.415	-0.3997	0.341	1.172	9
270	No	0.18861	0.9877	1.428	-0.44	0.3093	1.422	9
300	No	0.055567	0.8048	1.423	-0.6182	0.2813	2.197	9
