# Supplementary material for: Acetylcholine receptor based chemogenetics engineered for neuronal inhibition and seizure control assessed in mice
Source: Nat Commun. 2024 Jan 18;15:601. doi: 10.1038/s41467-024-44853-8 (PMC10796428; doi:10.1038/s41467-024-44853-8)
Supplement: Supplementary file 3 — Reporting Summary [file 41467_2024_44853_MOESM3_ESM.pdf]

nature portfolio

Corresponding author(s): Quynh-Anh Nguyen, Peter M. Klein

Last updated by author(s): Nov 29, 2023

Reporting Summary

Nature Portfolio wishes to improve the reproducibility of the work that we publish. This form provides structure for consistency and transparency in reporting. For further information on Nature Portfolio policies, see our Editorial Policies and the Editorial Policy Checklist.

Statistics

For all statistical analyses, confirm that the following items are present in the figure legend, table legend, main text, or Methods section.

n/a	Confirmed	
☐	☒	The exact sample size (*n*) for each experimental group/condition, given as a discrete number and unit of measurement
☐	☒	A statement on whether measurements were taken from distinct samples or whether the same sample was measured repeatedly
☐	☒	The statistical test(s) used AND whether they are one- or two-sided<br>*Only common tests should be described solely by name; describe more complex techniques in the Methods section.*
☐	☒	A description of all covariates tested
☐	☒	A description of any assumptions or corrections, such as tests of normality and adjustment for multiple comparisons
☐	☒	A full description of the statistical parameters including central tendency (e.g. means) or other basic estimates (e.g. regression coefficient) AND variation (e.g. standard deviation) or associated estimates of uncertainty (e.g. confidence intervals)
☐	☒	For null hypothesis testing, the test statistic (e.g. *F*, *t*, *r*) with confidence intervals, effect sizes, degrees of freedom and *P* value noted<br>*Give P values as exact values whenever suitable.*
☒	☐	For Bayesian analysis, information on the choice of priors and Markov chain Monte Carlo settings
☒	☐	For hierarchical and complex designs, identification of the appropriate level for tests and full reporting of outcomes
☒	☐	Estimates of effect sizes (e.g. Cohen's *d*, Pearson's *r*), indicating how they were calculated

Our web collection on statistics for biologists contains articles on many of the points above.

Software and code

Policy information about availability of computer code

Data collection	Zen Blue (Zeiss LSM 800 confocal microscope image acquisition); Matlab R2019b (seizure threshold testing, EEG recording, 2p imaging); pClamp 10 (in vitro and ex vivo electrophysiology)
Data analysis	Matlab R2019b, Pycharm Community Edition 2018.2.5, ImageJ 1.53, Graphpad Prism 9, Python 3.9.7, Pandas 1.3.4, Scipy 1.7.1, Statsmodels 0.13.2, Pingouin 0.5.2, Seaborn 0.12.1

For manuscripts utilizing custom algorithms or software that are central to the research but not yet described in published literature, software must be made available to editors and reviewers. We strongly encourage code deposition in a community repository (e.g. GitHub). See the Nature Portfolio guidelines for submitting code & software for further information.

Data

Policy information about availability of data

All manuscripts must include a data availability statement. This statement should provide the following information, where applicable:

- Accession codes, unique identifiers, or web links for publicly available datasets
- A description of any restrictions on data availability
- For clinical datasets or third party data, please ensure that the statement adheres to our policy

Plasmid sequences have been deposited into GenBank, accession number OR944653. Underlying datasets and analysis code that support the findings of this study have been deposited in the Zenodo database: https://doi.org/10.5281/zenodo.10223431

Research involving human participants, their data, or biological material

Policy information about studies with human participants or human data. See also policy information about sex, gender (identity/presentation), and sexual orientation and race, ethnicity and racism.

Reporting on sex and gender	N/A
Reporting on race, ethnicity, or other socially relevant groupings	N/A
Population characteristics	N/A
Recruitment	N/A
Ethics oversight	N/A

Note that full information on the approval of the study protocol must also be provided in the manuscript.

Field-specific reporting

Please select the one below that is the best fit for your research. If you are not sure, read the appropriate sections before making your selection.

☒ Life sciences ☐ Behavioural & social sciences ☐ Ecological, evolutionary & environmental sciences

For a reference copy of the document with all sections, see nature.com/documents/nr-reporting-summary-flat.pdf

Life sciences study design

All studies must disclose on these points even when the disclosure is negative.

Sample size	No statistical methods were used to determine sample size a priori. Sample size was determined based on preliminary experiments that enabled consistent group differences to be identified, and are indicated in figure legends.
Data exclusions	Whole cell data was excluded when predetermined parameters indicated poor recording quality. Animals with poor EEG signal or lack of observed seizures during baseline recordings were excluded.
Replication	For all in vivo experiments, replication was performed at least once and showed comparable results. The results from these experiments were pooled and used for analysis. In vitro recordings were replicated in samples from multiple distinct animals, with recordings from 1-6 cells per subject showing comparable results. Additionally, a subset of in vitro experiments were repeated by distinct researchers in a different facility and showed successful replications of experimental findings.
Randomization	Cell samples and mice were randomly assigned to each experimental group.
Blinding	Histology data was quantified by investigators blinded to the effect of the treatment. EEG data was quantified by investigators blinded to the identity of the expressed gene and drug treatment. In vitro electrophysiology recordings were performed without blinding to group allocation, as construct-expressing cells were identified by fluorescent tag expression. However, all electrophysiology analysis was performed algorithmically using software pipelines that were agnostic to group allocation. In vivo LFP recordings were performed in animals where group allocation was blinded and all analysis was performed algorithmically, independent of group identification. Group allocation of animals in seizure threshold testing experiments was blinded by assigning animals unique identification numbers not tied to their group identification.

Reporting for specific materials, systems and methods

We require information from authors about some types of materials, experimental systems and methods used in many studies. Here, indicate whether each material, system or method listed is relevant to your study. If you are not sure if a list item applies to your research, read the appropriate section before selecting a response.

Materials & experimental systems

n/a	Involved in the study	
☒	☐	Antibodies
☐	☒	Eukaryotic cell lines
☒	☐	Palaeontology and archaeology
☐	☒	Animals and other organisms
☒	☐	Clinical data
☒	☐	Dual use research of concern
☒	☐	Plants

Methods

n/a	Involved in the study	
☒	☐	ChIP-seq
☒	☐	Flow cytometry
☒	☐	MRI-based neuroimaging

Eukaryotic cell lines

Policy information about cell lines and Sex and Gender in Research

Cell line source(s)	AAV-293 HEK293 cells were obtained from Agilent.
Authentication	Further authentication of the commercially-obtained cell lines was not performed.
Mycoplasma contamination	Further mycoplasma contamination screening of the commercially-obtained cell lines was not performed.
Commonly misidentified lines (See ICLAC register)	N/A

Animals and other research organisms

Policy information about studies involving animals; ARRIVE guidelines recommended for reporting animal research, and Sex and Gender in Research

Laboratory animals	C57BL/6 mice (Jackson Labs Strain #664 for Stanford, Charles River Strain #27 for Coda, 3-8 week males or timed pregnant female). Animals were group housed under standard laboratory conditions (21°C ± 2°C; 50% ± 15% humidity).
Wild animals	Study did not involve wild animals.
Reporting on sex	Only male mice were used in the study, except in experiments utilizing cultured cells isolated from P0 animals where samples were pooled from multiple mice of varying sexes. Sex was not considered in the current study design and a sex-based analysis was not performed.
Field-collected samples	Study did not involve samples collected from the field.
Ethics oversight	All experimental protocols were approved by the Administrative Panel on Laboratory Animal Care of Stanford University or the Institutional Animal Care and Use Committee at Coda Biotherapeutics.

Note that full information on the approval of the study protocol must also be provided in the manuscript.

Plants

Seed stocks	N/A
Novel plant genotypes	N/A
Authentication	N/A
